# Identification and Mapping of Tomato Genome Loci Controlling Tolerance and Resistance to *Tomato Brown Rugose Fruit Virus*

**DOI:** 10.3390/plants10010179

**Published:** 2021-01-19

**Authors:** Avner Zinger, Moshe Lapidot, Arye Harel, Adi Doron-Faigenboim, Dana Gelbart, Ilan Levin

**Affiliations:** 1 The Institute of Plant Sciences, Agricultural Research Organization, Volcani Center, 68 HaMaccabim Road, P.O. Box 15159 Rishon LeZion 7505101, Israel; avner86@gmail.com (A.Z.); lapidotm@volcani.agri.gov.il (M.L.); aryeharel@volcani.agri.gov.il (A.H.); adif@volcani.agri.gov.il (A.D.-F.); danag@volcani.agri.gov.il (D.G.); 2 The Robert H. Smith Faculty of Agriculture, Food and Environment, The Hebrew University of Jerusalem, P.O. Box 12 Rehovot 7610001, Israel

**Keywords:** tomato, virus, resistance, ToBRFV, ToMV

## Abstract

Tomato brown rugose fruit virus (ToBRFV) was identified in Israel during October 2014 in tomato plants (*Solanum lycopersicum*). These plants, carrying the durable resistance gene against tomato mosaic virus, *Tm-2^2^*, displayed severe disease symptoms and losses to fruit yield and quality. These plants were found infected with a tobamovirus similar to that discovered earlier in Jordan. This study was designed to screen and identify tomato genotypes resistant or tolerant to ToBRFV. The identified resistance and tolerance traits were further characterized virologically and genetically. Finally, DNA markers linked to genes controlling these traits were developed as tools to expedite resistance breeding. To achieve these objectives, 160 genotypes were screened, resulting in the identification of an unexpectedly high number of tolerant genotypes and a single genotype resistant to the virus. A selected tolerant genotype and the resistant genotype were further analyzed. Analysis of genetic inheritance revealed that a single recessive gene controls tolerance whereas at least two genes control resistance. Allelic test between the tolerant and the resistant genotype revealed that these two genotypes share a locus controlling tolerance, mapped to chromosome 11. This locus displayed a strong association with the tolerance trait, explaining nearly 91% of its variation in segregating populations. This same locus displayed a statistically significant association with symptom levels in segregating populations based on the resistant genotype. However, in these populations, the locus was able to explain only ~41% of the variation in symptom levels, confirming that additional loci are involved in the genetic control of the resistance trait in this genotype. A locus on chromosome 2, at the region of the *Tm-1* gene, was finally found to interact with the locus discovered on chromosome 11 to control resistance.

## 1. Introduction

Viral diseases cause serious damage to plants by significantly reducing their yield and fruit quality. Plant viruses are mostly spread by insects, such as aphids, thrips and whiteflies, and are therefore one of the reasons why in many locations production has shifted from open field to protected environments [1]. The worldwide yield losses that can be ascribed to plant viruses are estimated to be more than 30 billion US$ annually [2]. 

One of the most devastating viruses infecting plants of the *Solanaceae* family and tomatoes in particular, are tobamoviruses. *Tobamovirus* is a genus in the *Virgaviridae* family that includes about 35 different virus species [3]. The two best-known viruses of this genus are tobacco mosaic virus (TMV) and tomato mosaic virus (ToMV) [4,5]. Unlike other viruses transmitted by vectors, *Tobamoviruses* are mechanically transmitted and are considered most persistent in terms of their ability to survive outside plant cells and in dead plant tissues [6].

For years, the main way to contain tobamoviruses was through preventative agro-technological means. These means including disinfection of agricultural areas and tools, rotation of seeds, use and replacement of detached soils, use of clean propagation material, and the removal of infected plants [4]. On the other hand, genetic resistance, if proven effective, is by far the preferred, economically sound and environmentally friendly way to prevent damage caused by the viruses [1].

Over the past 80 years, great advances have been made in our understanding of plant resistance against viruses. Approximately half of the known plant virus resistance genes are dominant [7]. In the last decade, a large number of crop recessive resistance genes were also identified. These resistances are often achieved through the absence of appropriate host factors required by the virus to complete its replication cycle [8]. Because plant viruses evolve, and at times acquire the ability to overcome resistance, the development of efficient and durable resistances, able to withstand the genetic plasticity of viruses, still represents a major challenge [8].

Two genes, *Tm-1* and *Tm-2*, conferring resistance to ToMV have been introgressed into the cultivated tomatoes (*Solanum lycopesicum*). The *Tm-1* gene, displaying a semi-dominant inheritance, was originally identified from *Solanum habrochites* [9,10]. This gene maps to the tomato chromosome 2 and encodes a ~80 kDa protein that physically binds to and functionally inhibits the replication proteins of ToMV [11]. The *Tm-2* resistance gene was discovered in *Solanum peruvianum* and found to confer a higher level of resistance compared to that displayed by *Tm-1*. The gene maps to the tomato chromosome 9 and harbors two resistant alleles: *Tm-2* and the *Tm-2^2^* [9,12], *Tm-2^2^* being more durable than *Tm-2* [13,14]. Consequently, *Tm-2^2^* is both practically and economically more important because it has been widely exploited as a ToMV resistance source in tomato breeding programs, and was found stable and effective for over 40 years. Both *Tm-2* and *Tm-2^2^* are dominant and encode a member of the CC-NBS-LRR class of resistance proteins [15].

Recently, a newly discovered tobamovirus that breaks down *Tm-2^2^* resistance was identified and named tomato brown rugose fruit virus (ToBRFV). The virus was identified in Jordan during 2015 [16]. A commercial tomato hybrid (*cv*. ‘Candela’), grown in greenhouses, showed mild foliar symptoms at the end of the season accompanied with strong brown rugose symptoms on fruits [16]. The causal agent was found to be transmitted mechanically to test plants that were later found to be positive to the virus. Following sequence comparisons with other tomato-infecting tobamoviruses, the new virus had the highest nucleotide sequence identity (82.4%) with the Ohio V strain of TMV. ToBRFV was first identified in a tomato greenhouse in southern Israel during 2014 on a number of different commercial tomato hybrids [17,18]. Tomato plants in this greenhouse, carrying the *Tm-2^2^* resistance gene, effective against ToMV, displayed disease symptoms that included a heavy mosaic pattern on leaves, narrowing of leaves, and yellow spotted fruit, causing heavy losses to fruit yield and quality. Within a short period of time, the new virus spread globally: during 2018 it was identified in tomato plants grown in Mexico, USA, Germany, Italy, and the Palestinian authority [19,20,21,22,23], and recently in Turkey, China, Greece, Egypt, and Spain [24,25,26,27,28]. This very rapid spread demonstrates that ToBRFV has become a worldwide threat to tomato production.

ToBRFV, found to overcome *Tm-2^2^* resistance, demonstrates the genetic plasticity of viruses in their interaction with resistance genes. This exemplifies the need for the continuous development of new, more efficient, and durable resistance genes able to withstand a wider range of virus strains, either alone or in combination with other resistances that were already identified.

This study was designed to screen and identify tomato genotypes resistant or tolerant to ToBRFV. The identified resistance and tolerance traits were further characterized virologically and genetically. Finally, DNA markers linked to genes controlling these traits were developed as tools to expedite resistance breeding.

## 2. Results

### 2.1. ToBRFV Overcomes Genetic Resistance to ToMV in Tomato

In Israel, ToBRFV was initially identified in commercial ToMV-resistant tomato plants carrying the *Tm-2^2^* gene [17]. To verify that ToBRFV can indeed infect ToMV-resistant plants, we have inoculated tomato open-pollinated genotypes, carrying either the *Tm-2* gene, the *Tm-2^2^* gene, or a combination of *Tm-1* and *Tm-2^2^.* The plants were inoculated with either ToMV or with ToBRFV and compared with an open-pollinated genotype carrying no ToMV-resistance gene. Results presented in Table 1 show that genotypes resistant to ToMV displayed no disease following inoculation with ToMV. In contrast, the ToMV-resistant genotypes displayed very high average disease severity index (DSI) and viral levels when infected with ToBRFV, very much like the ToMV-susceptible control.

### 2.2. Screening for Tomato Genotypes Resistant or Tolerant to ToBRFV

At least eight plants of each one of 160 genotypes were initially screened in a greenhouse with ToBRFV (total number of plants >1280). At least eight plants of each genotype showing no symptoms were inoculated with ToBRFV again to validate their phenotype. Of the 160 genotypes screened, 29 (18.1%) were found tolerant to ToBRFV. Plants of these tolerant genotypes showed no symptoms following inoculation with ToBRFV but were characterized with viral levels that were as high as those in susceptible genotypes. Of these 29 tolerant genotypes, nine (31.0%) belong to *Solanum pimpinellifolium* and eight (27.6%) were cultivated lines or hybrids. 

In contrast to our success in identifying tolerant genotypes, we have managed to identify only a single ToBRFV-resistant genotype. Plants of this genotype showed no symptoms and extremely low viral levels following inoculation with ToBRFV (average DSI = 0.0 ± 0.1, average viral level = 0.0 ± 0.0). This resistant genotype (VC554) and a representative tolerant genotype (VC532; average DSI = 0.1 ± 0.0, average viral level = 724 ± 52) were selected for further studies. The tolerant genotype was selected because it displayed the most consistent phenotype following several rounds of inoculations.

### 2.3. Genetic Inheritance of ToBRFV-Tolerance in VC532

A total of 104 F_2_ plants of the initial cross between the tolerant VC532 genotype and a susceptible genotype LA2706 [Moneymaker without *Tm-2^2^* (Table 1)], together with the parental lines and their F_1_ hybrid plants were inoculated with ToBRFV to evaluate DSI. Results, presented in Table 2, show that while the tolerant line VC532 displayed very low average DSI levels, its F_1_ crossbred plants with the susceptible line displayed very high average DSI levels that did not differ from the susceptible line. This indicates that the tolerance trait is controlled in a recessive manner. Of the 104 F_2_ plants inoculated, 25 (24%) showed no symptoms, similar to the tolerant parent, indicating that a single recessive gene controls tolerance [χ^2^ = 0.05, *P*(χ^2^) = 0.8].

### 2.4. Genetic Inheritance of ToBRFV-Resistance in VC554

A total of 160 F_2_ plants of the initial cross between the resistant VC554 genotype and the susceptible genotype LA2706 together with the parental lines and their F_1_ hybrid plants were inoculated with ToBRFV to evaluate DSI. Results, presented in Table 3, show that while the resistant line VC554 displayed no symptoms at all, its F_1_ crossbred plants with the susceptible line displayed DSI levels that were in between those exhibited by the resistant and the susceptible lines (i.e., 1.9). This indicates that the resistance trait is controlled in a partially dominant manner. Of the 160 F_2_ plants inoculated, 64 (40%) showed no symptoms, similar to the resistant parent, whereas the rest showed DSI values ranging from 1 to 3. These results cumulatively suggest that at least two genes control resistance in VC554 and that at least one of them is either dominant or semi-dominant.

### 2.5. Allelic Test between VC532 and VC554

To test whether the tolerant genotype VC532 shares genetic components controlling ToBRFV symptoms with the resistant genotype VC554, we have carried out an allelic test between these two genotypes. For that, we crossed the two parental lines to obtain F_1_ plants that were self-pollinated to acquire an F_2_ population of 222 plants. Following inoculation with ToBRFV, the two parental lines, their F_1_ crossbred plants, and F_2_ plants were all found symptomless (Table 4). However, only the resistant VC554 plants were characterized with an extremely low average ToBRFV level, significantly differing from all other genotypes in the analysis. These results indicate that VC532 and VC554 share the gene (or genes) controlling ToBRFV tolerance, whereas resistance is controlled by an additional gene (or genes). It is noteworthy that nearly 25% of the F_2_ plants were found to be with no virus.

### 2.6. Mapping-by-Sequencing of the Locus Controlling Tolerance

**Figure 1:**
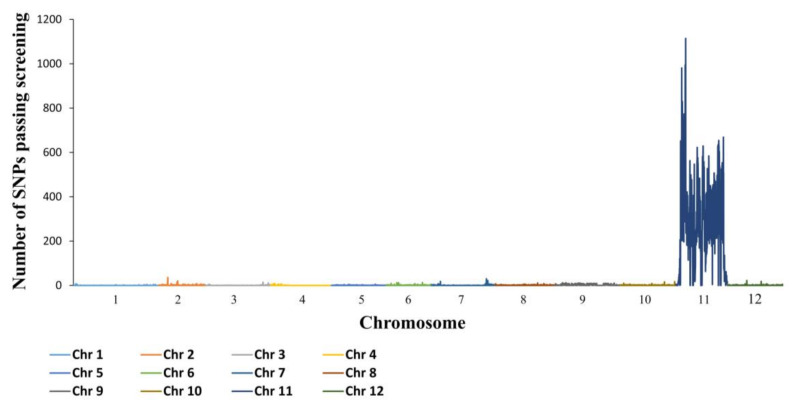
Mapping-by-sequencing of the gene controlling tolerance in VC532 [Each chromosome (Chr) is marked by a different color].

In an effort to identify and map the quantitative trait locus (QTL) controlling toler-ance, DNA pools extracted from susceptible and tolerant F_2_ plants, resulting from a cross between VC532 and a susceptible Moneymaker genotype, carrying the *Tm-2^2^* gene (LA3310, Table 1), as well as DNA samples extracted from their two parental lines, were subjected to High Throughput Sequencing (HTS). The variant calling procedure yielded ~5.8 million single nucleotide polymorphisms (SNPs) and insertion-deletions (INDELs). Further screening of these polymorphisms, as detailed in the Materials and Methods section, led to the identification of 184,401 SNPs. Of these 184,401 SNPs, 140,583 (76.2%) were mapped to chromosome 11, while the rest were scattered throughout the entire genome. These results, presented in Figure 1, point only to chromosome 11 as the one carrying the QTL controlling tolerance. The wide spread of this QTL on chromosome 11 will be dissected in our discussion.

### 2.7. Development of DNA Markers for the Analysis of Tolerance in VC532

A set of eleven sequence-characterized amplified region (SCAR) DNA markers, scattered throughout chromosome 11, were developed for the analysis of association with the tolerance trait in VC532 (Table 5). Analysis of segregating populations, resulting from initial crosses between VC532 and susceptible genotypes, revealed that although all of these markers were significantly associated with the tolerance, the *BstNI_*8.89 marker exhibited the highest level of association and therefore was used in most of our further analyses.

### 2.8. Analysis of Association between the BstNI_8.89 Marker and Tolerance in VC532

An F_2_ population of 168 plants resulting from a cross between VC532 and the susceptible genotype Moneymaker (LA2706), together with their respective control lines, were inoculated with ToBRFV and their symptoms recorded (Table 6). The F_2_ plants were genotyped with *BstNI_*8.89 and a highly significant association between this marker and DSI was obtained [*P*(F) = 1.5 × 10^−85^, R^2^ = 91%, Table 6].

### 2.9. Analysis of Association between the BstNI_8.89 Marker and DSI in VC554

An F_2_ population of 168 plants resulting from a cross between VC554 and the susceptible genotype LA2706 (Moneymaker with no ToMV resistance gene, Table 1), together with their respective control lines, were inoculated with ToBRFV and their symptoms recorded. The F_2_ plants were genotyped with *BstNI_*8.89 and a highly significant association between this marker and DSI was obtained (Table 7). However, relative to the segregating population based on the tolerant VC532 line, the *BstNI_*8.89 marker effect in the segregating population based on VC554 was significantly lower [*P*(F) = 3.1 × 10^−19^] with a much lower coefficient of determination (R^2^ = 41%). The statistically significant association between the *BstNI_*8.89 marker and DSI and its recessive nature, presented in Table 7, confirm the allelic test between VC532 and VC554 (Table 4), indicating that VC532 and VC554 share a recessive locus controlling DSI on chromosome 11. The significantly lower effect of *BstNI_*8.89 and its much lower R^2^, obtained in the segregating population based on the resistant VC554 genotype, suggest that additional loci may control DSI in VC554 and, based on the comparisons between VC532, the susceptible LA2706 line and their F_1_ plants (Table 7), these loci should exert a semi-dominant effect. 

### 2.10. Association between the Tm-1 Locus and ToBRFV Resistance in VC554 

In an effort to identify additional loci that may affect DSI and viral level in VC554, we carried out HTS analysis on DNA extracts from VC554 and directly compared them with sequences of susceptible lines. As expected, high level of nucleotide variation was found on chromosome 11, similarly to that obtained in the analysis of VC532 (Figure 1). In addition, a high level of nucleotide variation was observed on chromosome 2 at the vicinity of the *Tm-1* gene. Of 364,410 SNPs discovered, 202,097 (55.5%) were found on chromosome 11, whereas 58,931 (16.2%) were found on chromosome 2, mostly centered at the vicinity of the *Tm-1* gene. The rest of the SNPs were scattered throughout the remaining genome. DNA sequences obtained from VC554, coupled with direct sequencing of cDNA synthesized from VC554 with primers complementary to the *Tm-1* gene sequence, revealed that VC554 carries the ToMV-resistant allele of the *Tm-1* gene (GenBank accession GCR237) and not its cultivated susceptible counterpart *tm-1* (GenBank accession GCR26).

A DNA marker that differentiates between lines carrying the resistant *Tm-1* gene sequence and their cultivated counterpart was developed. The marker was used to analyze the F_2_ population presented in Table 7 in a two-way analysis, together with *BstNI_*8.89, and their one-way interaction. This analysis increased the coefficient of determination (R^2^) from 41% to 59%, and all sources of variation were found statistically significant: *P*(F) = 8.9 × 10^−20^ for *BstNI_*8.89, 10.0 × 10^−8^ for *Tm-1*, and 2.6 × 10^−3^ for *BstNI_*8.89 × *Tm-1* interaction. In this analysis, *BstNI_*8.89 displayed a recessive effect, similar to that presented in Table 6, whereas *Tm-1* displayed a semi-dominant effect (heterozygous *Tm-1/tm-1* plants displaying average DSI that was statistically lower than homozygous *tm-1/tm-1* plants and statistically higher than homozygous *Tm-1/Tm-1* plants).

The significant main effect of the *Tm-1* locus was surprising because our earlier results show that plants carrying both *Tm-1* and *Tm-2^2^* in a homozygous state were highly susceptible to ToBRFV (Table 1). To solve this discrepancy, we carried out two different experiments. In the first experiment, the following genotypes were inoculated with ToBRFV: four different accessions carrying *Tm-1* in homozygous state, two *Tm-2*/*Tm-2* genotypes, three genotypes carrying both *Tm-1* and *Tm-2* in homozygous state, and two genotypes with no ToMV resistance gene. Results presented in Table 8 show that all genotypes displayed the highest possible average DSI (3.0), irrespective of the ToMV resistance gene or gene combination they carry. These results also suggest that *Tm-1* alone is not directly involved with a reduction in ToBRFV symptoms, nor is its combination with *Tm-2*. For the second experiment, the F_2_ population developed for the allelic test presented in Table 4 was genotyped with two DNA markers. One marker was based on the *Tm-1* gene region, and another was *TaqI_*8.71 that maps at close vicinity to *BstNI_*8.89 (Table 5). The *TaqI_*8.71 marker, developed to differentiate between VC532 and susceptible genotypes, was also able to differentiate between VC532 and VC554. Using these two markers, we were able to select plants with the following six genotypes: (1) *11^VC532^/11^VC532^ Tm-1/Tm-1*, (2) *11^VC532^/11^VC532^ tm-1/tm-1*, (3) *11^VC554^/11^VC554^*, *Tm-1/Tm-1*, (4) *11^VC554^/11^VC554^ tm-1/tm-1*, (5) *11^VC532^/11^VC554^ Tm-1/Tm-1,* and (6) *11^VC532^/11^VC554^ tm-1/tm-1*. Two plants of each one of these six genotypes were self-pollinated to obtain F_3_ seeds. Eight F_3_ plants, originating from each one of these plants were inoculated with ToBRFV, their symptoms were recorded, and their viral level was quantified. As expected, results show that no symptoms were displayed by the two parental lines as well as by the F_3_ plants (Table 9). The symptomless phenotype of all plants in this experiment can clearly be attributed to the tolerance QTL on chromosome 11, shared by all plants. The average ToBRFV viral levels and their range, however, point to an apparent interaction between this QTL and a QTL at the *Tm-1* region on chromosome 2. F_3_ plants, carrying only the QTL on chromosome 11, had high ToBRFV levels. On the other hand, plants that carry this QTL in combination with the QTL on chromosome 2, marked by the *Tm-1* marker, had significantly lower viral levels that were not statistically remote from 0.0. These results show that ToBRFV resistance in VC554 is attributed to the interaction between the two QTLs, and that resistance can also be obtained by combining the tolerance QTL discovered in VC532 with the QTL marked by *Tm-1* on chromosome 2.

### 2.11. The Tm-2 Locus Is Not Associated with ToBRFV Tolerance or Resistance

Results presented in Table 1 and Table 8 show that plants carrying either *Tm-2* or *Tm-2^2^* are highly susceptible to ToBRFV, and so are plants carrying each one of these two *Tm-2*-gene alleles in combination with *Tm-1*. This suggests that the *Tm-2* locus, on its own, is not associated with ToBRFV tolerance or resistance. The ineffectiveness of *Tm-*2 locus in controlling ToBRFV symptoms can be also deduced from our mapping-by-sequencing results presented in Figure 1; whereas the susceptible genotype used in this analysis was homozygous for *Tm-*2^2^, no effect on symptoms was obtained at the location of the *Tm-2* gene on chromosome 9. It can, however, be argued that the *Tm-2* locus may exert its effect via an interaction with the tolerance QTL on chromosome 11 or via a three-way interaction with this QTL and the *Tm-1* locus. To resolve this argument, we first examined the DNA sequences we obtained from VC532 and VC554 and confirmed that they do not carry either one of the *Tm-2* alleles. In addition, we generated a large F_2_ population originating from a cross between VC554, carrying the tolerance QTL on chromosome 11 and the *Tm-1* resistance gene, and the Moneymaker *cv.*, carrying the stronger *Tm-2^2^* allele (LA3310). These F_2_ plants were inoculated with ToBRFV and showed that the *Tm-2^2^* locus did not have any effect on symptom development; alone [*P*(F) = 0.10], in combination with the *Tm-1* locus [*P*(F) = 0.15], in combination with the tolerance QTL on chromosome 11 [*P*(F) = 0.11] and in a three-way combination with these two loci [*P*(F) = 0.42].

## 3. Discussion

ToBRFV is an emerging, fast-spreading, tobamovirus that causes significant losses to tomato yield and its fruit quality. In this manuscript, we clearly demonstrate that *Tm-1* and *Tm*-*2*, two genes known to control resistance to ToMV, are not solely effective in controlling ToBRFV infection in tomato plants. This includes the more durable *Tm*-*2^2^* allele of the *Tm*-*2* gene that was found stable and effective in controlling ToMV for more than 40 years [13]. We also demonstrate that combinations of *Tm-1* with either one of the two alleles of the *Tm-2* gene are ineffective in controlling ToBRFV as well. The failure of these interactions to yield ToBRFV resistant plants may be surprising because each one of these two genes controls ToMV infection through a different route. While *Tm-1* encodes a ~80 kDa protein that physically binds to and functionally inhibits the replication proteins of ToMV [11], both *Tm-2* and *Tm-2^2^* encode a member of the CC-NBS-LRR class of resistance proteins that inhibits viral cell-to-cell movement [15].

The lack of available sources of resistance to ToBRFV justifies our effort to screen ToBRFV-resistant or -tolerant genotypes. Our screening included 160 tomato genotypes that do not represent the entire repertoire of the tomato clade because we have chosen to first screen genotypes more closely related to the cultivated tomato. We speculated that, if successful, this approach could substantially simplify and expedite the introgression of QTLs controlling ToBRFV-resistance or -tolerance into the parental lines of elite tomato genotypes. Our screening resulted in an unexpectedly high percentage of tolerant genotypes with nearly 28% of them being cultivated lines or hybrids. These results suggest that the tolerance trait is simply inherited (i.e., controlled by few genes), or that a positive breeding value can be attributed to genes controlling tolerance or to genes tightly linked to them. In contrast to our success in identifying tolerant genotypes, we have managed to identify only a single ToBRFV-resistant genotype. The rare occurrence of ToBRFV-resistant genotypes in our sample of genotypes may justify screening of additional genotypes, in particular those genetically distant from the cultivated tomato. In addition, this rare occurrence may hint to a more complex genetic inheritance of ToBRFV resistance relative to that of the tolerance trait. Overall, our results demonstrate that the tomato gene pool, represented in this study by only 160 genotypes, can serve as a resource for identifying genotypes that are either tolerant or resistant to ToBRFV.

A representative tolerant genotype (VC532) and the resistant genotype (VC554) were subjected for further studies. Our genetic inheritance studies showed that a single recessive locus controls tolerance VC532, while at least two loci control resistance in VC554, at least one of them being either dominant or semi-dominant. Furthermore, a comprehensive allelic test carried out between VC532 and VC554 showed that these two genotypes share the gene (or genes) controlling tolerance, while resistance is controlled by an additional gene (or genes).

Mapping-by-sequencing, carried out on segregating populations derived from a cross between VC532 and a susceptible genotype, identified chromosome 11 as the one carrying genes controlling tolerance (Figure 1). The wide spread of the QTL on chromosome 11 may indicate either that more than a single gene on chromosome 11 control tolerance in VC532, or point to a compression of segregation on this chromosome. Although we cannot, currently, rule out that more than a single gene on chromosome 11 is controlling tolerance in VC532, analysis of DNA markers scattered throughout the entire chromosome (Table 5) indicated that the segregation on chromosome 11 is compressed, particularly in regions downstream from *BstNI_*8.89 towards the end of the chromosome. For example, nine recombinants were found between *PvuI*_7.52 and *BstNI_*8.89 in an F_2_ population of 166 plants, indicative of a recombination frequency of 1.98 Centimorgan (cM) per 1 Mbp, whereas only 11 recombinants were found between *BstNI_*8.89 and *EcoRI*_50.82, indicative of a recombination frequency of 0.08 cM per 1 Mbp, nearly 25 times lower. This compression resulted in a statistically significant association between each one of the DNA markers presented in Table 5 and DSI in this F_2_ population. Fine-tune mapping of the tolerance QTL, utilizing recombinant plants, should finally resolve this matter. 

A highly significant association was obtained between *BstNI_*8.89 and DSI in an F_2_ segregating population obtained from the initial cross between VC532 and a susceptible genotype [*P*(F) = 1.5 × 10^−85^, R^2^ = 91%, Table 6]. Because VC532 and VC554 were found to share the tolerance locus, we expected that a significant association would be also obtained between *BstNI_*8.89 and DSI in an F_2_ segregating population obtained from the initial cross between VC554 and a susceptible genotype. We also expected that this association would be lower than that obtained for VC532 because the genetic inheritance of VC554 indicated that more than a single gene might be involved in ToBRFV resistance in this line. Indeed, the *BstNI_*8.89 marker effect in the segregating population was significant but lower than its effect on in the segregating population based on VC532 [*P*(F) = 3.1 × 10^-19^] with a much lower R^2^ (41%).

In an effort to identify additional loci that can possibly affect DSI and viral levels in VC554, we carried out HTS analysis of DNA extracted from VC554 and directly compared with results obtained from susceptible lines. As expected, a QTL was discovered on chromosome 11, similar to that revealed in the analysis of the tolerant VC532 (Figure 1). In addition, a QTL was discovered on chromosome 2, at the vicinity of the *Tm-1* gene, that led us further to find that VC554 carries the resistant allele of this gene. A DNA marker developed based on the *Tm-1* gene region, indicated that *Tm-1* is associated with DSI and interacts with *BstNI_*8.89. This was surprising because our results did not point to the *Tm-1* region as a possible locus that may affect ToBRFV symptoms on its own or in combination with either one of the two alleles of the *Tm-2* gene. We therefore speculated that the interaction between the *Tm-1* region and *BstNI_*8.89 on chromosome 11 is the main cause for symptom reduction and resistance in the VC554 line. Analysis of F_3_ plants of the initial cross between VC532 and VC554 finally confirmed that resistance to ToBRFV could be obtained by combining the *BstNI_*8.89 QTL, mapped to chromosome 11, either from VC532 or VC554, with the *Tm-*1 region on chromosome 2, inherited in this research from VC554. However, it is not currently clear whether *Tm-1* itself is participating in controlling the resistance or rather genes linked to it. Finally, we also show that the *Tm-2* gene does not affect ToBRFV resistance, either on its own or in combination with the other two loci presented in this manuscript.

Our results show that tolerance to ToBRFV was exclusively mapped to chromosome 11, validating its simple genetic inheritance as a single recessive gene. Although we cannot currently confirm whether a single gene controls tolerance, such a possibility is intriguing because tolerance is considered to be a complex genetic trait that involves multiple molecular mechanisms operating simultaneously [29].

The resistance trait discovered was found to be the product of a genetic interaction between the tolerance locus on chromosome 11 and a locus situated at the *Tm-1* region on chromosome 2, whereas *Tm-1* alone had no effect on ToBRFV infection. Although we cannot currently confirm whether the *Tm-1* gene itself participates in this interaction, this possibility is not completely remote because this gene has been implicated in ToMV-resistance and can thus serve as an excellent candidate gene [11,14].

The participation of tolerance in the resistance phenotype may be advantageous, harnessing the benefits of tolerance, compared to resistance, in terms of reduced selection pressure of emerging virulent isolates, increased breadth and stability of the phenotype, and potential benefits to the host, as exemplified in natural environments [29].

Although natural sources of tolerance traits are available for some economically important crops, they are generally poorly characterized [29]. The discovery of the locus controlling tolerance to ToBRFV on chromosome 11 will permit better characterization of tolerance trait, following fine-tune mapping and map-based cloning of the causative genes.

## 4. Materials and Methods

### 4.1. Plant Material and Resource Populations 

Seeds of tomato genotypes were obtained from the Tomato Genetics Resource Center (TGRC) at the University of California, Davis, CA, USA (https://tgrc.ucdavis.edu/), from seed stocks available at the Volcani Center and from seed stocks obtained from the laboratory of Professor Dani Zamir, the Hebrew University, Rehovot, Israel. These genotypes included cultivated commercial hybrids or their selections in advanced generations; wild tomato species, in particular those closely related to the cultivated tomato; genotypes displaying amino-acid variation at the *Tm-2* locus (based on the genomic browser available at https://solgenomics.net/jbrowse_solgenomics), and genotypes with wild introgressions at the *Tm-1* or the *Tm-2* locus. At least eight plants of 160 genotypes were inoculated with ToBRFV and eight of these genotypes were separately inoculated with ToMV.

Two genotypes were chosen for in-depth study: a tolerant genotype and a genotype resistant to the virus (VC532 and VC554, respectively). Both genotypes were each crossed to the susceptible *S. lycopersicum cv.* Moneymaker (LA2706) and VC554 was also crossed to *S. lycopersicum cv.* Moneymaker carrying the *Tm-2^2^* gene (LA3310). The resultant F_1_ plants were allowed to self-pollinate to obtain segregating F_2_ populations. In addition, VC532 and VC554 were cross-pollinated and their resultant F_1_ plants were self-pollinated to obtain F_2_ seeds.

### 4.2. Virus Maintenance, Virus Acquisition and Plant Inoculation

ToBRFV (GeneBank Acc. No. KXG619418) was maintained on Moneymaker tomato plants carrying the *Tm-2^2^* (LA3310) while ToMV was maintained on Moneymaker (LA2706) plants in an insect-proof greenhouse. The cultures were propagated and renewed every three-to-four weeks by mechanical inoculation. The virus was transmitted mechanically: leaves of ToBRFV-infected tomato source plants were ground in 0.01 phosphate buffer (pH 7.0) and applied to carborundum dusted test plants. The carborundum was washed out and the test plants were kept in a temperature-controlled greenhouse (18/25 °C Min/Max) under natural conditions without artificial light. All inoculations were carried out in 8-row × 16-column sowing trays with 40 mL planting soil (Hishtil Plant-Nursery Company, Israel). Inoculations were carried out at the first true leaf stage of the seedlings, approximately two weeks after sowing. Seedlings were fertilized on a weekly basis throughout the experiment. ToBRFV-induced symptoms and viral levels were evaluated using the procedures described below.

### 4.3. Disease Severity Scoring

ToBRFV- or ToMV-induced symptoms were evaluated 30 days post inoculation (DPI), and at times later, in a temperature controlled greenhouse. The symptoms were evaluated according to the disease severity index (DSI): (0) no visible symptoms, inoculated plants show the same growth and development as non-inoculated plants; (1) light mosaic pattern on the apical leaf; (2) severe mosaic pattern on the apical leaf, (3) very severe mosaic pattern, coupled with pronounced elongation or folding of the apical leaf.

### 4.4. Enzyme-Linked Immunosorbent Assay (ELISA) to Evaluate Viral Levels

Indirect ELISA analyses were performed on plant leaves using laboratory-produced specific antibodies against ToMV or ToBRFV (a kind gift from Dr. A. Dombrovsky, ARO, Rishon LeZion, Israel) as previously described [30,31]. In the analysis, two discs, 1 cm in diameter, taken from the 4th and the 5th true leaf represented each plant. Samples were taken 30 days after inoculation, ground in coating buffer (Agdia) and incubated for 3 h at 37 °C with 1:5000 dilution of the specific laboratory-produced antiserum (anti-ToBRFV or anti-ToMV). Detection was carried out by incubating the samples with AP-conjugated goat anti-rabbit (IgG) (Sigma, Steinheim, Germany) for 3 h at 37 °C. P-nitro phenyl phosphate (Sigma) substrate was used at a concentration of 0.6 mg/mL. The developing color was recorded by Multiskan FC microplate photometer (Thermo Fisher Scientific, Waltham MA, USA) at 405 nm and 620 nm. Optical density (OD) values of a minimum ratio of three times the value of the negative, non-infected controls were considered positive.

### 4.5. Genomic DNA Extraction and Analysis of Markers by Polymerase Chain Reaction (PCR)

Genomic DNA was extracted from individual plants according to Fulton et al. [32]. PCR primers were designed using the Primer3 software version 4.0 (https://bioinfo.ut.ee/primer3-0.4.0/). The PCRs were performed in a volume of 15 μL containing 10 ng of template DNA, 10 pmol of each of two primers, 0.2 mM of each dNTP, 2 mM MgCl_2_, 1 U of Taq DNA polymerase, and 1XPCR-buffer. The PCRs conditions were: 94 °C 3 min, followed by 35 cycles of 94 °C for 30 s, 58–60 °C for 30 s (depending on primers’ characteristics), and 72 °C for 1 min. Final elongation was at 72 °C for 10 min. Amplification products were digested with restriction endonucleases and visualized by electrophoresis in 2% agarose gel. Primer sequences for the *Tm-1* gene locus were (5’-to-3’): F-TCTCACCATTCTCACACTGAGTTAC and R-ACTGAAGGAAACAATACCAAGTCTG. The PCR fragment obtained by these primers was digested by *Eco147I* to differentiate between genotypes carrying the resistant and the susceptible allele. Primer sequences for the *Tm-2* gene locus were (5’-to-3’): F-TACAAACCTTGATGTGGATACCTG and R-CACAGCAACGTGAGTGTAGTAGTG. The PCR fragment obtained by these primers was digested by *ApaLI* to differentiate between genotypes carrying the resistant and the susceptible allele. Sequences of the other primers used in this study are presented in Table 5 and in Table 10.

### 4.6. RNA Extraction and Sequencing of the Tm-1 Gene in VC554

Total RNA was extracted from 25 mg of leaf tissue of VC554 plants using the TRI-reagent (Sigma-Aldrich, St. Louis, MO, USA) and DNA contaminants were digested with TURBO DNA-free DNAase (Ambion, Austin, TX, USA). Total RNA was used as the template for first-strand cDNA synthesis using the Superscript pre-amplification system (Gibco BRL Life Technologies, Gaithersburg, MD, USA) according to the manufacturer’s instructions. Briefly, 5 μg of total RNA was transcribed in a reaction mix containing 15 pmol of gene-specific primers, 4 μg reaction buffer, 1 μg RNase inhibitor, 2 μL of 10 mM dNTP mix, and 1 μl of reverse transcriptase (200 U/μL). The reaction mix volume was adjusted to 20 μL by adding nuclease free water. The 20 μl mix was incubated in 42 °C for 60 min and then heated to 70 °C for 5 min to terminate the reaction. This cDNA obtained was used as a template in PCR reactions to amplify seven overlapping fragments of the gene encoding *Tm-1* in VC554 with primers presented in Table 10. PCR amplification conditions were identical to those presented in Section 4.5. The PCR products were directly sequenced by Ornat Laboratories (Nes Ziona, Israel). Sequences obtained were viewed and scanned using the SnapGene software (https://www.snapgene.com/snapgene-viewer/). Validated sequences were finally compared to the ToMV-resistant allele of the gene (GenBank accession GCR237) and to its cultivated susceptible counterpart *tm-1* (GenBank accession GCR26) using the Basic Local Alignment Search Tool (https://blast.ncbi.nlm.nih.gov/Blast.cgi).

### 4.7. Mapping-by-Sequencing of the Gene Controlling Tolerance 

To map the gene controlling tolerance, we followed the procedure presented by Soyk et al. [33]. However, unlike Soyk et al. [33], we present the number of SNPs passing the three careening criterions outlined below rather than SNP ratio (Figure 1). For this mapping analysis, an F_2_ population was generated by crossing the tolerant VC532 with the susceptible Moneymaker genotype carrying the *Tm-2^2^* gene (LA3310). From 335 ToBRFV-inoculated F_2_ plants, we selected 25 plants displaying the most severe symptoms (DSI = 3.0), and 25 plants displaying no visible symptoms, but virus infected as confirmed by ELISA. DNA was extracted from each one of these plants using the DNeasy Plant Mini Kit (QIAGEN, https://www.qiagen.com/us/). 0.4 µg of DNA was taken to represent each plant in the susceptible and the symptomless DNA pool. In addition, six µg of DNA were extracted from each one of the two parental lines. HTS of the four DNA samples, including the libraries preparation, were carried out in Cornell University, Ithaca, NY, USA as described by Soyk et al. [33] with the following modification: ~100 bp paired-ends sequencing reads were obtained using the Illumina HighSeq2000 machine (San Diego, CA, USA).

On average, 125,012,108 (~40 Gb) reads were obtained. The FASTX Toolkit (http://hannonlab.cshl.edu/fastx_toolkit/) was used for: (1) trimming read-end nucleotides with quality scores <30 using fastq_quality_trimmer; (2) removing reads with a quality score ≤30 using fastq quality filter. ~91% of an average total of 94,657,458 cleaned reads, obtained after processing and cleaning, were successfully mapped onto the SOL reference genome database available at https://solgenomics.net/, version Sol 3.0, using the Burrows-Wheeler Aligner (BWA) software 0.7.12-r1039, with its default parameters [34]. The resulting mapping file was processed using SAMtools/Picard tool (http://broadinstitute.github.io/picard/, version 1.78); [35] for adding read group information, sorting, marking duplicates and indexing. Then, the local realignment process for locally realigning reads was performed so that the number of mismatching bases is minimized across all the reads using the RealignerTargetCreator and IndelRealigner of the Genome Analysis Toolkit version 3.4-0 (GATK; version http://www.broadinstitute.org/gatk/); [36]. Finally, the variant calling procedure was performed using HaplotypeCaller of the GATK toolkit (https://gatk.broadinstitute.org/hc/en-us) developed by Broad Institute of MIT and Harvard (Cambridge, MA, USA). Only sites with DP (read depth) higher than 20 were analyzed.

To detect nucleotides associated with the tolerance trait, we applied the following criterions: (1) such nucleotides should be in a homozygous state in the tolerant parent and identical in the tolerant F_2_ DNA pool, because the tolerance trait was found to be recessive (as detailed in our results); (2) such nucleotides should be polymorphic between the tolerant and the susceptible parents (homozygous nucleotides in the tolerant parent should be replaced by a different nucleotide in a homozygous state in the susceptible parent because the two lines are open-pollinated); and (3) homozygous nucleotides in the tolerant parent should be replaced by a different nucleotide in a homozygous state or be in heterozygous state in the susceptible DNA pool. These criterions are based on the hypothesis that the tolerant parents and the tolerant F_2_ bulk are expected to share common regions of the genome that are linked with ToBRFV tolerance. These linked regions should be different (missing and alternate) in the susceptible parent and the susceptible F_2_ bulk. 

### 4.8. Quantitative and Qualitative Statistical Analyses

Viral level and DSI recorded at 30 DPI, or later, for each plant as well as the association between DNA markers and DSI, were assessed by analyses of variance. The genetic inheritance of the tolerance and the resistance traits were evaluated by Chi-square (χ^2^). All analyses were carried out with the JMP Pro 15 statistical discovery software (SAS Institute Inc., Cary, NC, USA). Differences among means are presented as different superscript letters that represent statistically significant mean values (*P* < 0.05) based on Tukey-Kramer Honestly Significant Difference (HSD) test [37]. 

## 5. Conclusions

ToBRFV is an emerging, fast-spreading, tobamovirus that causes significant losses to tomato yield and its fruit quality. The virus was found to overcome known tobamovirus resistance genes, in particular the *Tm-2^2^* gene that, for decades, effectively controlled ToMV infection, underlying the genetic plasticity of tobamoviruses. 

Our results demonstrate that the available tomato gene pool, represented in this study by only 160 genotypes, can serve as a resource for identifying genotypes that are either tolerant or resistant to ToBRFV. These traits were further characterized and genetically analyzed to provide a set of DNA markers that can expedite their introgression into elite tomato cultivars. These markers will also provide the tools for our on-going effort to identify the genes controlling tolerance or resistance to ToBRFV. The identification of these genes is expected to expand our understanding of the molecular mechanisms controlling the interaction between plants and viruses.

## Figures and Tables

**Table 1:** Average disease severity index (DSI) and average viral level in tomato mosaic virus (ToMV)-resistant and susceptible tomato plants infected with ToMV or with tomato brown rugose fruit virus (ToBRFV).

			DSI		Viral Level (OD)	
*S. lycopersicum cv.*	ToMV Resistance Gene	N	ToMV	ToBRFV	ToMV	ToBRFV
Moneymaker (LA2706)	-	8	2.6 ^A^ ± 0.1	2.7 ^A^ ± 0.1	647 ^A^± 10	802 ^A^ ± 25
T-5 (LA2399)	*Tm-2*	8	0.3 ^B^ ± 0.0	2.8 ^A^ ± 0.1	11 ^B^± 1	731 ^A^ ± 18
Momor (LA2828)	*Tm-2^2^*	8	0.0 ^B^ ± 0.0	3.0 ^A^ ± 0.0	6 ^B^ ± 1	396 ^B^ ± 5
Vendor (LA2968)	*Tm-2^2^*	8	0.0 ^B^ ± 0.0	2.9 ^A^ ± 0.1	5 ^B^ ± 1	733 ^A^ ± 22
Moneymaker (LA3310)	*Tm-2^2^*	8	0.0 ^B^ ± 0.0	3.0 ^A^ ± 0.0	20 ^B^ ± 2	1072 ^A^ ± 30
Mocimor (LA2830)	*Tm-1, Tm-2^2^*	8	0.4 ^B^ ± 0.0	2.4 ^A^ ± 0.2	6 ^B^ ± 1	659 ^A^ ± 19

Results are presented as Mean ± Standard Error, viral level was determined by ELISA using specific antibodies for each tested virus and is presented as optical-density (OD) × 1000, N denotes number of plants, and different superscript letters above means express a statistically significant difference, *P* < 0.05, based on Tukey-Kramer Honestly Significant Difference (HSD) test.

**Table 2:** Average ToBRFV disease severity index (DSI) of the tolerant genotype (VC532), the susceptible genotype (Moneymaker), their F_1_ hybrid and F_2_ plants.

Genotype	N	DSI
Moneymaker LA2706	8	3.0 ^A^± 0.0
VC532	8	0.2 ^B^ ± 0.2
F_1_ (Moneymaker × VC532)	8	3.0 ^A^ ± 0.0
F_2_ (Moneymaker × VC532)	104	2.2 ^A^ ± 0.1

N denotes number of plants, results are presented as Mean ± Standard Error, and different superscript letters above means express a statistical significant difference, *P* < 0.05, based on Tukey-Kramer Honestly Significant Difference (HSD) test.

**Table 3:** Average ToBRFV disease severity index (DSI) of the resistant genotype (VC554), the susceptible genotype (Moneymaker), their F_1_ hybrid and F_2_ population.

Genotype	N	DSI
Moneymaker LA2706	8	3.0 ^A^ ± 0.0
VC554	8	0.0 ^C^ ± 0.0
F_1_ (Moneymaker × VC554)	8	1.9 ^AB^ ± 0.2
F_2_ (Moneymaker × VC554)	160	1.3 ^BC^ ± 0.1

N denotes number of plants, results are presented as Mean ± Standard Error, and different superscript letters above means express a statistically significant difference, *P* < 0.05, based on Tukey-Kramer Honestly Significant Difference (HSD) test.

**Table 4:** Allelic test between the tolerant genotype VC532 and the resistant genotype VC554.

Genotype	N	DSI	DSI Range	ToBRFV Level (OD)	ToBRFV-Level Range (OD)
VC532	8	0.0 ^A^ ± 0.0	0.0–0.0	1072 ^A^ ± 185	721–1486
VC554	8	0.0 ^A^ ± 0.0	0.0–0.0	0 ^B^ ± 6	0–3
F_1_ (VC532 × VC554)	8	0.0 ^A^ ± 0.0	0.0–0.0	1200 ^A^ ± 227	768–1617
F_2_ (VC532 × VC554)	222	0.0 ^A^ ± 0.0	0.0–0.5	766 ^A^ ± 70	0–3016

Average disease severity index (DSI) and average ToBRFV viral level are presented as Mean ± Standard Error, ToBRFV viral levels are presented as optical-density (OD) × 1000, N denotes number of plants, and different superscript letters above means express a statistical significant difference, *P*(F) < 0.05, based on Tukey-Kramer Honestly Significant Difference (HSD) test.

**Table 5:** Primers used to obtain sequence-characterized amplified region (SCAR) DNA markers for the association studies on chromosome 11.

Marker Name *	5’-to-3’ Forward (F) and Reverse (R) Primer Sequences
*PvuI*_7.52	F-GGGAGAATTGAATGTGGAGGGT
	R-GACAGGTTCGTGATTGCAGC
*DraI*_8.04	F-GCAGATTTAGAGGTCAGATCCTTC
	R-CATGCCAGTACCAGAGTTCAATAG
*EcoRI_*8.38	F-TCACTCTCAAAGCAATTCATAATGT
	R-GAGCTTCAGTGGGTCTCAAT
*TaqI_*8.71	F-ATCCTACCAACTCCGAGAATAGAAC
	R-GGTGTGGAATCTAGCAACATAAATC
*BstNI_*8.89	F-GGTACCCTCTCAATCTCAAGGTC
	R-GAATTTACACGCCACCTTCCTC
*Bsp119I_*9.28	F-TCGAGAGAGGAGAGGTTATAAGGAC
	R-GACGGGGTATTCCTTGGTTATC
*Eco105I_*9.34	F-AGAACTACTGCCTCGAGTTTCTTC
	R-ACTCTCGAGTCTAGACACTCATTGG
*DraI_*9.58	F-GAAGAAGAAGCAGGCCAGAAAG
	R-GAGATAGCCGAGCGATATTGAG
*HhaI_*9.78	F-TCCAAGTGGCATGTTTAATGAC
	R-CGAGTTCCAATACTTTCCCATC
*BcuI*_9.94	F-GTCGTGACAGAAATAGATGGAATG
	R-TACATCTGACCCTCATATGCTAGG
*EcoRI* *_50.82*	F-CAGAGAGTTACACACGACCAAGTC
	R-AGGTTATCTTCTTTAACCCCCAAG

* Marker name is composed of the restriction endonuclease used to digest the PCR products and its approximate map location in Mbp on chromosome 11.

**Table 6:** Analysis of association between the *BstNI_8.89* marker and ToBRFV disease severity index (DSI) in F_2_ plants originating from an initial cross between the susceptible genotype LA2706 and the tolerant VC532 genotype.

Parental Lines and F_1_ Plants			Analysis of Association		
Genotype	N	DSI	*BstNI_8.89*	N	DSI
Moneymaker (LA2706)	8	3.0 ^A^ ± 0.0	SS	44	2.9 ^A^ ± 0.0
VC532	8	0.0 ^B^± 0.2	ST	67	2.6 ^A^ ± 0.1
F_1_ (Moneymaker × VC532)	8	2.9 ^A^ ± 0.1	TT	57	0.0 ^B^ ± 0.0

The parental lines and their F_1_ crossbred plants are presented in the left portion of the table whereas the analysis of association in F_2_ plants is presented in the right part. *BstNI_8.89* genotypes: SS represent homozygous plants with the marker variant inherited from the susceptible parent, TT represent homozygous plants with the marker variant inherited from the tolerant genotype, and ST represent heterozygous plants. N denotes number of plants, results are presented as Mean±Standard Error, and different superscript letters above means express a statistically significant difference, *P* < 0.05, based on Tukey-Kramer Honestly Significant Difference (HSD) test.

**Table 7:** Analysis of association between the *BstNI_8.89* marker and ToBRFV disease severity index (DSI) in F_2_ plants originating from an initial cross between the susceptible genotype LA2706 and the resistant VC554 genotype.

Parental Lines and F_1_ Plants			Analysis of Association		
Genotype	N	DSI	*BstNI_8.89*	N	DSI
Moneymaker (LA2706)	8	3.0 ^A^± 0.0	SR	83	2.0 ^A^ ± 0.1
VC554	8	0.0 ^C^± 0.0	SS	41	1.8 ^A^± 0.3
F_1_ (Moneymaker × VC554)	8	1.9 ^B^± 0.2	RR	44	0.4 ^B^± 0.1

The parental lines and their F_1_ crossbred plants are presented in the left portion of the table whereas the analysis of association in F_2_ plants is presented in the right part. *BstNI_8.89* genotypes: SS represent homozygous plants with the marker variant inherited from the susceptible parent, RR represent homozygous plants with the marker variant inherited from the resistant genotype, and SR represent heterozygous plants. N denotes number of plants, results are presented as Mean ± Standard Error, and different superscript letters above means express a statistically significant difference, *P* < 0.05, based on Tukey-Kramer Honestly Significant Difference (HSD) test.

**Table 8:** Average disease severity index (DSI) of different Tomato Genetics Resource Center (TGRC) accessions carrying *Tm-1*, *Tm-2*, a combination of *Tm-1* and *Tm-2*, or no ToMV resistance gene following inoculation with ToBRFV.

Accession	Genotype	N	DSI
LA2706	*cv.* Moneymaker	8	3.0 ^A^ ± 0.0
LA2838A	*cv.* Ailsa Craig	8	3.0 ^A^ ± 0.0
LA2825	*Tm-1/Tm-1* in *cv.* Moneymaker background	8	3.0 ^A^ ± 0.0
LA3269	*Tm-1/Tm-1* in *cv.* Ailsa Craig background	8	3.0 ^A^ ± 0.0
LA3271	*Tm-1/Tm-1* in *cv.* Ailsa Craig background	8	3.0 ^A^ ± 0.0
LA3276	*Tm-1/Tm-1* in *cv.* Ailsa Craig background	8	3.0 ^A^ ± 0.0
LA3268	*Tm-2*/*Tm-2* in *cv.* Ailsa Craig background	8	3.0 ^A^ ± 0.0
LA2399	*Tm-2*/*Tm-2* in *cv.* T5 background	8	3.0 ^A^ ± 0.0
LA3297	*Tm-1/Tm-1*, *Tm-2*/*Tm-2* in *cv.* Vagabond background	8	3.0 ^A^ ± 0.0
LA3432	*Tm-1/Tm-1, Tm-2/Tm-2* in *cv.* Ailsa Craig background	8	3.0 ^A^ ± 0.0
LA3812	*Tm-1/Tm-1, Tm-2/Tm-2* in *cv.* Ailsa Craig background	8	3.0 ^A^ ± 0.0

Results are presented as Mean ± Standard Error, N denotes number of plants, and identical superscript letters above means express no statistical significant difference, *P* > 0.05, based on Tukey-Kramer Honestly Significant Difference (HSD) test.

**Table 9:** Average disease severity index (DSI) and average ToBRFV level and its range in parental lines (upper part of the table) and in F_3_ gene combinations inoculated with ToBRFV (lower part of the table).

	Genotype	N	DSI	ToBRFV Level (OD)	ToBRFV-Level Range (OD)
**Parental Lines**	VC532	8	0.0 ^A^ ± 0.0	834.1 ^A^ ± 62.0	600–900
	VC554	8	0.0 ^A^ ± 0.0	5.0 ^B^ ± 1.9	0–12
	*11^VC532^/11^VC532^ Tm-1/Tm-1*	16	0.0 ^A^ ± 0.0	5.1 ^B^ ± 1.9	0.0–26.9
	*11^VC554^/11^VC554^ Tm-1/Tm-1*	16	0.0 ^A^ ± 0.0	3.8 ^B^ ± 1.8	0.0–22.9
**F_3_ Plants**	*11^VC532^/11^VC554^ Tm-1/Tm-1*	16	0.0 ^A^ ± 0.0	0.0 ^B^ ± 0.0	0.0–0.0
	*11^VC532^/11^VC532^ tm-1/tm-1*	16	0.0 ^A^ ± 0.0	619.8 ^A^ ± 36.3	323.3–838.7
	*11^VC554^/11^VC554^ tm-1/tm-1*	16	0.0 ^A^ ± 0.0	498.7 ^A^ ± 42.9	208.5–978.3
	*11^VC532^/11^VC554^ tm-1/tm-1*	16	0.0 ^A^ ± 0.0	544.1 ^A^ ± 47.5	242.8–1334.5

Results are presented as Mean±Standard Error, average viral levels and viral-level range are presented as optical-density (OD) × 1000, N denotes number of plants, and different superscript letters above means express a statistically significant difference *P* < 0.05, based on Tukey-Kramer Honestly Significant Difference (HSD) test.

**Table 10:** Primers used to amplify overlapping fragments of the *Tm-1* gene in the resistant VC554 genotype.

Primer name	Primer Sequence
*Tm_1_A_F*	CCTCTCCACTTGACGGTTGT
*Tm_1_A_R*	TCTGCAAACGTGCCCATAGTT
*Tm_1_B_F*	GAGATCCAGTCTTAACAGCTTCTCC
*Tm_1_B_R*	ACTTTCCATTAGTGATGCTATGCTC
*Tm_1_C_F*	TTGCCAGTGGTCAAACTGAA
*Tm_1_C_R*	ACGCATTAGGGAAACCTGCT
*Tm_1_D_F*	ACGACAACTGAGGTTGCAGA
*Tm_1_D_R*	TCTACGTGTCTAGATTTCGGAGAGA
*Tm_1_E_F*	ACGTTGTCAGGTTAAGGTCCTCC
*Tm_1_E_R*	TGGAAAGTTTTGCACCCCACA
*Tm_1_F_F*	CTTGTACAACTCAGGGCGCTT
*Tm_1_F_R*	ACACTTTCCTCCAATGAGACGG
*Tm_1_G_F*	AGTTGCTCATATGGGGCTTACA
*Tm_1_G_R*	GCAGTGCTGTAGTGTTTCAAGTT

## Data Availability

Not applicable.
